# Evaluation of prognostic factors for mortality in cancer patients with sepsis in the intensive care unit: systematic review protocol

**DOI:** 10.62675/2965-2774.20250283

**Published:** 2025-04-22

**Authors:** María Fernanda García-Aguilera, Nayely García-Méndez, Glenn Hernández, Borja M. Fernández-Félix, Harold Alexander-León, Yunqi Yu-Liu, Josue Rivadeneira, Luis Fuenmayor-González, Cristopher Isaac Peña Robayo, Fernanda Villalba, Eduardo Andrés Aragundi Palacios, Emérita Eugenia Basantes Borja, Henry Caballero Narvaez, Isabel Morales Alcocer, Eduardo Velazco, Georgina Muñoz, Juan Pablo Holguín-Carvajal, Tamara Otzen Hernández, Carlos Manterola

**Affiliations:** 1 Universidad de La Frontera Temuco Chile Universidad de La Frontera - Temuco, Chile.; 2 Universidad Central del Ecuador Faculty of Medical Sciences Quito Ecuador Faculty of Medical Sciences, Universidad Central del Ecuador - Quito, Ecuador.; 3 Hospital Oncológico SOLCA Quito Ecuador Hospital Oncológico SOLCA, Núcleo de Quito - Quito, Ecuador.; 4 Hospital Eugenio Espejo Quito Ecuador Hospital Eugenio Espejo - Quito, Ecuador.; 5 Pontificia Universidad Católica de Chile School of Medicine Department of Intensive Care Medicine Santiago Chile Department of Intensive Care Medicine, School of Medicine, Pontificia Universidad Católica de Chile - Santiago, Chile.; 6 Hospital Universitario Ramón y Cajal Clinical Biostatistics Unit Madrid Spain Clinical Biostatistics Unit, Hospital Universitario Ramón y Cajal - Madrid, Spain.; 7 CIBER Epidemiology and Public Health Madrid Spain CIBER Epidemiology and Public Health - Madrid, Spain.; 8 Zero Biomedical Research Quito Ecuador Zero Biomedical Research - Quito, Ecuador.; 9 Universidad Católica de Santiago de Guayaquil Guayaquil Ecuador Universidad Católica de Santiago de Guayaquil - Guayaquil, Ecuador.

**Keywords:** Sepsis, Neoplasms, Prognostic, Risk factors, Mortality, Systematic review protocol

## Abstract

**Introduction:**

This systematic review outlines a comprehensive approach to identify and analyze prognostic factors associated with mortality in adult cancer patients with sepsis in the intensive care unit. The review will focus on all-cause 28-day mortality, and where not available, we will use 30-day, intensive care unit, or in-hospital mortality.

**Methods and analysis:**

We present a protocol for the systematic review of prognostic factors for mortality in adult cancer patients with sepsis managed in the intensive care unit. Our primary outcome is 28-day mortality, and where not available, we will use 30-day, intensive care unit, or in-hospital mortality. The secondary outcome is the global mortality incidence. Studies on the basis of the population (sepsis and neoplasms), prognostic study methods and outcome of interest (mortality) will be included. We will search the following databases: Medline, PubMed, EMBASE, SCOPUS, Web of Science, and Bireme-BVS, until April 5, 2024. The risk of bias will be assessed using the QUIPS tool. A meta-analysis will be conducted where possible to generate pooled estimates for identified prognostic factors. Two authors will independently assess the risk of bias in each study using the Quality in Prognostic Studies tool. The GRADE approach will be employed to evaluate the overall quality of evidence and the strength of the recommendations. Findings will be disseminated through publication in a peer-reviewed journal. This review aims to provide clinicians with valuable insights into factors influencing mortality risk in this high-risk population, ultimately informing clinical decision-making and improving patient outcomes.

**Ethics and socialization:**

The results of this review will be published in a peer-reviewed scientific journal. Does not require ethical approval.

## INTRODUCTION

The sustained increase in cancer incidence globally poses significant challenges, not only in the management of cancer itself but also in the management of associated severe complications such as sepsis, which is especially prevalent and fatal in this patient population.^(1,2)^ Medical advances have improved cancer survival; however, susceptibility to severe infections such as sepsis remains high due to the immunosuppression induced by the oncological disease and its treatments.^(2-4)^

Cancer patients develop sepsis at an alarming rate and are more likely to be admitted to intensive care units (ICUs), which are associated with high mortality rates.^(2)^ This not only represents a clinical problem but also a considerable economic burden for health systems owing to the extensive resources these patients require.^(5)^

Given this context, it is essential to identify and improve the understanding of the risk factors that contribute to mortality in adult cancer patients with sepsis in the ICU for the future development of strategies that will enhance outcomes and reduce mortality rates. Previous studies have suggested that patient-related and clinical management factors significantly influence outcomes.^(6,7)^ However, variability in study designs and methodological quality has prevented definitive conclusions, highlighting the need for a rigorous and structured systematic review that consolidates and critically evaluates the existing evidence.

## METHODOLOGY

### Record

The protocol has been registered in accordance with the recommendations of the protocol statement of the Preferred Reporting Items for Systematic Review and Meta-Analysis (PRISMA) in the International Prospective Register of Systematic Reviews (PROSPERO) on November 23, 2023.^(8)^ The systematic review will be reported following the PRISMA initiative.^(9)^

### Eligibility criteria

#### Type of study

Experimental and observational studies that report prognostic/risk factors for mortality in adult cancer patients with sepsis and septic shock treated in the ICU will be included.

#### Types of participants

For a study to be eligible, participants must be adults 18 years of age and older with a diagnosis of cancer and sepsis admitted to the ICU. The study will be required to include information from studies that have reported information on any of the main outcomes of ICU, hospital or 28–30-day mortality. We will include studies published from 2004 onward and perform a stratified meta-analysis by decade to evaluate differences in outcomes between studies published in the first decade (2004 - 2013) and second decade (2014-2024). The definitions of Sepsis-1, Sepsis-2, and Sepsis-3 will be accepted, as well as the definitions provided by the International Classification of Diseases and the Centers for Disease Control and Prevention (CDC).^(10-13)^

#### Types of outcomes

### Primary outcomes

Our primary outcome is 28-day mortality, and where not available, we will use 30-day, ICU, or in-hospital mortality.

### Secondary outcomes

Global mortality incidence (the most extended follow-up provided by the study authors).

### Information sources

#### The following databases will be searched:

- MEDLINE
- EMBASE
- Web of Science (WoS)
- Scopus
- Bireme-BVS

#### Types of prognostic factors

We will include all studies that evaluate any prognostic factors for mortality, and the associations between the detected prognostic factors and the outcomes of patients with sepsis in the ICU will subsequently be assessed.

#### Core adjustment set

**Figure 1:**
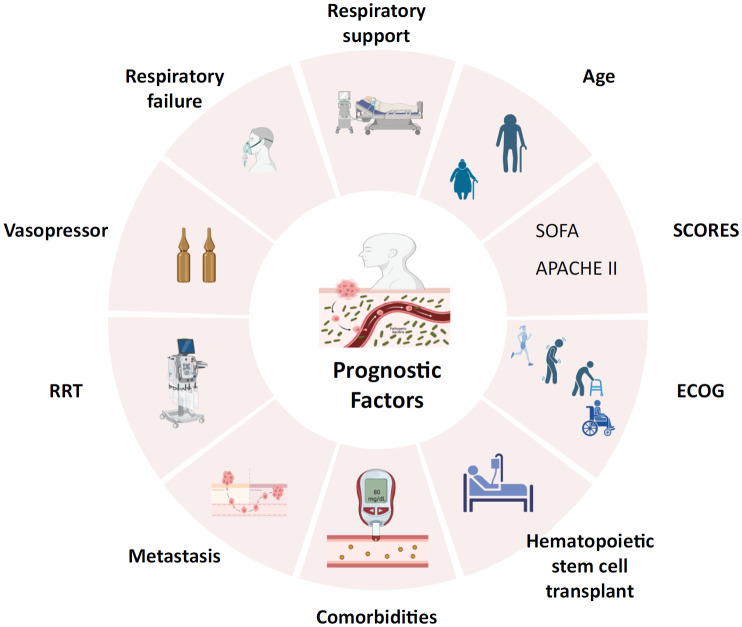
Prognostic factors of mortality in cancer patients with sepsis in the intensive care unit.

We will assess the role of the identified factors after adjustment for additional covariables. After a consensus among experts, studies will be considered if they have adjusted for at least one of the following factors: 1) sex; 2) age; 3) type of tumor (locoregional solid tumor, metastatic solid tumor, or hematological); 4) severity score (severity score; Sequential Organ Failure Assessment score [SOFA], Eastern Cooperative Oncology Group [ECOG]); 5) comorbidities (type 2 diabetes mellitus, chronic heart failure, chronic pulmonary disease [COPD]; 6) support in the ICU (ventilator support, use of vasopressor, renal replacement therapy, hospital origin, transfusions, chemotherapy); 7) complications (neutropenia, atrial fibrillation, acute renal failure, infections due to viruses, bacteria, fungi and parasites, and respiratory failure); and 8) hematopoietic stem cell transplant (HSCT), recipients for a more accurate mortality assessment than other HSCT types or nontransplanted individuals^(14-16)^ (Figure 1). If new evidence is found, these parameters will be modified.

Created in BioRender.com

APACHE II - Acute Physiology and Chronic Health Evaluation; ECOG - Eastern Cooperative Oncology; RRT - renal replacement therapy; SOFA - Sequential Organ Failure Assessment.

#### Search methods

The search strategy will be based on terms related to populations (sepsis; neoplasms), prognostic factor (prognostic factors), outcome (mortality), and predictive study methods,^(17)^ without time and language limits.

#### Study data management

We will remove duplicate articles and select articles using the Covidence program, followed by manual cross-checking.^(18)^ The resulting unique references will be manually filtered for additional duplicate checking. The nonduplicated data will then be selected to construct the data extraction drafts and extracted.

#### Study selection and data collection

Two of the researchers will independently review the documents in Covidence according to best practice guidelines to evaluate the inclusion criteria. If there are discrepancies, a third researcher will resolve them. The inclusion criteria will be assessed in a sample of studies to ensure reproducibility. Two researchers will independently extract the data via a predefined form, and a third researcher will resolve any discrepancies. Three studies will be piloted on the data extraction template to ensure suitability. The results will be compared, and a third researcher will resolve any discrepancies.

#### Data elements

The checklist for critical appraisal and data extraction for systematic reviews of prediction modeling studies for prognostic factor (CHARMS-PF) will be used for critical evaluations and data extraction.^(19,20)^ For each eligible study, we will extract the following data:

- General information: author, publication date, number of patients, place (country), and financing of the studies included
- Source of data: prospective or retrospective design
- The authors’ definitions of "sepsis" and "septic shock"
- Participant characteristics: clinical and demographic data, eligibility criteria and recruitment criteria
- Outcome(s): type of mortality (ICU, hospitalization, and/or at 28 – 30 days), definition and timing
- Missing data, if applicable: number of participants with missing data on outcome and prognostic factors and methods applied for handling missing data
- Statistical analysis: logistic regression model, Cox regression model, machine learning, neural networks, or other methods
- Prognostic factor selection: a method to select prognostic factors (all prognostic factors on the basis of prior knowledge or on the basis of univariable associations)
- Confounder factor selection: method for selecting adjustment confounders: prespecified model, retrospective elimination, prospective selection, horizontal selection, LASSO-based selection, ridge-type regression, or bootstrap-type selection
- Estimates reported between the prognostic factor and each outcome: (1) unadjusted estimate: association between sepsis prognostic factors and mortality without any covariate; (2) adjusted approaches: associations between and mortality with at least one covariate from the selected core set
- Type of measure of association: odds ratio (OR)

#### Assessment of methodological quality and risk of bias

To evaluate the quality and biases of the original articles, the Cochrane methodology and QUality In Prognostic Studies (QUIPS) tool will be used,^(21,22)^ considering that the format is accepted as one of the most precise and useful tools for systematic reviews.^(23,24)^ The domains of the QUIPS will be evaluated independently by two authors, and in the case of discrepancy, they will be resolved by a third author. The selection of studies by inclusion and content evaluation will be conducted with clear, predefined criteria through systematic data collection. Studies will be independently examined on the basis of a set of preestablished criteria to establish the validity of the study.

#### Data synthesis

For each study and prognostic factor, we will extract the measures of association (overall mortality, ICU, hospital, and 28–30-day mortality) together with their confidence intervals (CIs) or standard errors (SEs). To allow statistical pooling of the estimates, we will transform the measures of association into ORs with 95%CIs. We consider that the association measures and their adjustments may differ from the results. We will develop a set of key-adjusted and unadjusted factors to review each result.

If the adjusted factors match with our groups established for the review, we will proceed with a meta-analysis of the article to penalize the estimates of the results in the evaluation of the risk of bias. If the study presents different estimates for the same result, we will extract the estimation that includes the maximum number of confounding factors. If there are multiple approximations that adjust for the same confounding factors, we will consider only the approximation adjusted for the maximum number of confounding factors to minimize the risk of bias due to confounding factors in the approach. The results of each study will be tabulated using Microsoft Excel V. 16. and the prebuilt CHARMS-PROBAST format.^(21)^

We plan to combine the results from individual studies in a meta-analysis to provide a pooled effect estimate for each prognostic factor and outcome. We will aggregate the data via a DerSimonian-Laird random-effects restricted maximum likelihood (REML) meta‐analysis model providing pooled estimates and Knapp-Hartung 95%CIs, and between-study variance estimates in Stata (Version 18) will be used.^(25)^ If we cannot obtain pooled estimations, we will describe the results in a narrative way.

**Figure 2:**
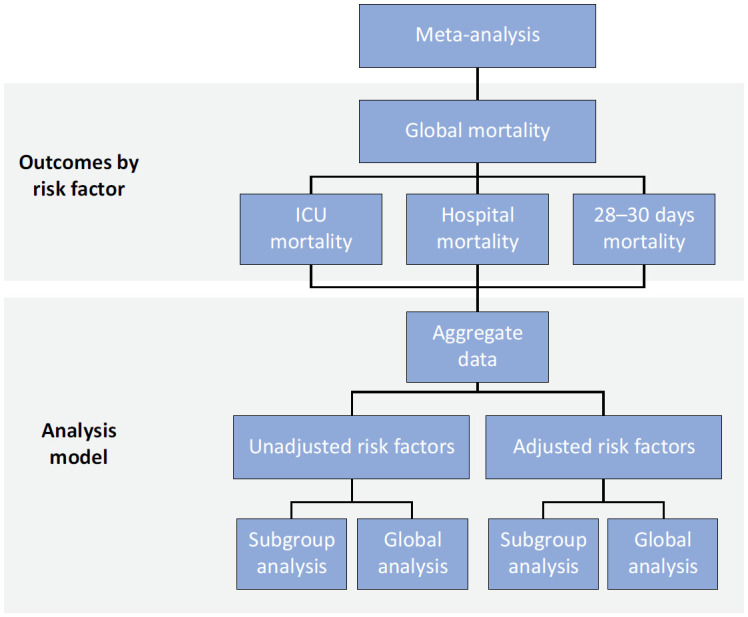
Analysis model.

Finally, the prevalence of mortality will be examined in proportions. We will aggregate the data using a random‐effects model using the inverse Freeman–Tukey transformation. We will carry out subgroup analysis by type of malignancy (solid tumors and hematologic malignancies) and geographical area (i.e., continent) (Figure 2).

Created in BioRender.com

ICU - intensive care unit.

#### Heterogeneity between studies

Heterogeneity will be examined when there are at least two articles, and the prediction interval will be calculated to report the variation in effects between populations.

#### Sensitivity analysis

Through sensitivity analysis, the robustness and stability of the results obtained will be evaluated against the methodology and study inclusion criteria. This analysis will also help identify potential biases and the influence of individual studies on generalizable conclusions.

The sensitivity analysis will exclude studies that present a high risk of bias, according to the criteria established in the "Quality Assessment of Studies" section. This will allow us to determine whether studies with lower methodological quality significantly affect the results of the meta-analysis so that they can be excluded according to quality.

Sensitivity analyses will be considered within specific subgroups (e.g., cancer type, patient age) to examine whether these variables may influence the relationship between prognostic factors and mortality in this population. This is to identify whether the associations vary significantly between different population groups or according to different conditions.

#### Publication bias

To detect the presence of publication bias in our review, the funnel plot method will be used, where the effect sizes of individual studies will be plotted against their precision (inverse standard error). An asymmetric distribution of research in the graph will suggest the possible existence of publication bias.

We will complement the graphical analysis with statistical tests, including the Egger test to evaluate asymmetry in the funnel plot through an intercept regression and the Begg test for the correlation between the ranges of estimated effects and their variances.^(26)^ A significant result in these tests will indicate a possible presence of publication bias.

If publication bias is detected, we will apply adjustment techniques such as the "trim and fill" method, which estimates the number of missing studies in the funnel plot and adds them to correct the estimated combined effect. This method will help provide a tighter estimate of the true effect, considering publication bias.

We will evaluate the limitations that publication bias could impose on the interpretation of our findings. To ensure that users of our systematic review understand the potential influence of publication bias on the confidence and applicability of the results.

#### Confidence in cumulative evidence

We will apply the Grading, Development and Evaluation of Recommendations (GRADE) approach^(27)^ and present each main result in a table, following the principles of Hughes et al. for prognostic questions, considering that the GRADE approach has been widely used to evaluate the certainty of evidence for prognostic factors.^(28)^

#### Patient and public participation

The public and patients will not participate in this systematic review.

#### Dissemination and ethics

Approval from an ethics committee is not required when summarizing already published data. The study will be coordinated by the Universidad Central del Ecuador, Hospital Oncológico SOLCA, Núcleo de Quito—Research Department. We will publish and disseminate our results through a peer-reviewed indexed journal, at conferences, and in the media.
